# Effects of insurance status on children's access to specialty care: a systematic review of the literature

**DOI:** 10.1186/1472-6963-7-194

**Published:** 2007-11-28

**Authors:** Asheley Cockrell Skinner, Michelle L Mayer

**Affiliations:** 1 Department of Health Policy and Administration, University of North Carolina at Chapel Hill, Chapel Hill, North Carolina, USA; 2 Cecil G. Sheps Center for Health Services Research, University of North Carolina at Chapel Hill, Chapel Hill, North Carolina, USA

## Abstract

**Background:**

The current climate of rising health care costs has led many health insurance programs to limit benefits, which may be problematic for children needing specialty care. Findings from pediatric primary care may not transfer to pediatric specialty care because pediatric specialists are often located in academic medical centers where institutional rules determine accepted insurance. Furthermore, coverage for pediatric specialty care may vary more widely due to systematic differences in inclusion on preferred provider lists, lack of availability in staff model HMOs, and requirements for referral. Our objective was to review the literature on the effects of insurance status on children's access to specialty care.

**Methods:**

We conducted a systematic review of original research published between January 1, 1992 and July 31, 2006. Searches were performed using Pubmed.

**Results:**

Of 30 articles identified, the majority use number of specialty visits or referrals to measure access. Uninsured children have poorer access to specialty care than insured children. Children with public coverage have better access to specialty care than uninsured children, but poorer access compared to privately insured children. Findings on the effects of managed care are mixed.

**Conclusion:**

Insurance coverage is clearly an important factor in children's access to specialty care. However, we cannot determine the structure of insurance that leads to the best use of appropriate, quality care by children. Research about specific characteristics of health plans and effects on health outcomes is needed to determine a structure of insurance coverage that provides optimal access to specialty care for children.

## Background

In recent years, access to pediatric specialty care has become a concern in the United States. While much of this concern stems from a known maldistribution and assumed shortage of these providers [1], insurance status is another important determinant of access. In the current economic climate, many states facing budget shortfalls are seeking ways to reduce expenditures in their Medicaid and State Children's Health Insurance (SCHIP) programs, including reducing covered services, increasing copayments, limiting those eligible for coverage [2], and implementing enrollment freezes in SCHIP [3]. Families with private insurance are also struggling, as employers turn to lower-cost plans with reduced benefits and increased cost-sharing, require greater employee contributions to premiums, particularly for family coverage, or offer catastrophic-only coverage [4].

These changes in the availability and benefit structures of health insurance could detrimentally affect access to specialty care. Uninsured adults and those in health maintenance organizations (HMOs) are significantly less likely than privately insured persons to use specialty care, and those covered by Medicaid are the least likely of all to use specialty care [5]. In contrast, other studies found that managed care enrollees receive more referrals from their primary care physician than other adults [6] and that removal of gatekeeping mechanisms does not increase use of specialty care [7].

The effects of insurance on children's access to specialty care may differ from the effects that have been noted in adult populations. For example, the supply-side effects of insurance status are less certain for pediatric specialty care as pediatric subspecialists are heavily concentrated in academic medical centers [8,9]. Here managed care arrangements may indirectly influence the number of pediatric subspecialists by changing institutional staffing plans [10]. In addition, fewer than 5% of providers in any given pediatric specialty are located in HMOs [9], which may limit access for these children despite having insurance coverage. The demand for specialty services also differs markedly between pediatric and adult populations. With a few notable exceptions, chronic pediatric conditions are relatively rare, leading to less demand for these providers relative to their internal medicine counterparts.

Although previous research demonstrates a strong relationship between insurance status and primary care access, these findings may differ for pediatric specialty care. Pediatric specialists, with their heavy concentration in academic medical centers, may be less sensitive to price than primary care physicians. Consequently, commonly-held differences between private insurance, Medicaid, and self-pay patients may not be as strong for children's specialty care. Coverage for primary care needs is common across private insurance, Medicaid, and SCHIP plans whereas coverage for specialty care varies more widely, often with greater copayments and more stringent definitions of medical necessity in private insurance plans. More generous specialty coverage by Medicaid may minimize differences between public and private coverage in access to subspecialty care.

On the other hand, access to specialty care may be more complex than access to primary care, magnifying the effects of insurance status for a child. In most cases, children are referred to specialty care from a primary care physician, which may serve as an additional barrier to accessing and using specialty care, particularly for children who are uninsured. Also, specialty care is generally more expensive, whether paid out of pocket or with greater insurance copayments, which may have an even greater negative effect on children who are uninsured or low-income insured. Among privately insured children, gatekeeping arrangements or restrictions on referrals to out of network providers may constrain access.

To develop a more complete picture of access to pediatric specialty care, it is important to understand how insurance status affects access to and actual use of this care. While specialty care is often required of children with special health care needs, it is not limited to this group, as children without chronic medical needs may also need access to specialty care. To this end, we performed a literature review to summarize the effects of insurance on children's access to specialty care.

## Methods

### Search Protocol

This review is part of a broader review of access to and quality of specialty care for children that has been previously described [11]. We used Pubmed to search for all articles related to our question of interest. Because of inconsistencies in the assignment of MeSH terms to articles, we used an exhaustive list of MeSH terms to identify articles (Additional File 1). Each individual specialty was searched for relevant articles and all articles were cross-referenced with children or pediatrics.

### Eligibility Criteria

To be retained for the literature synthesis, each article had to 1) present original data, 2) study children or adolescents or physicians involved in their care, 3) address the effects of insurance on access to or use of specialty care, and 4) be performed in the United States.

We included English-language articles published between January 1, 1992 and July 31, 2006. We excluded editorials, comments, letters, review articles and meta-analyses, practice guidelines, and policy statements. Because we were interested in physician care of children with medical problems requiring specialty care we excluded articles on dentistry, nursing, and primary care, acute illness (such as upper respiratory infections), immunizations, reproductive health and prenatal care. In addition, we excluded all studies of mental health issues, because insurance for mental health coverage often differs markedly from coverage for other specialty care. We also excluded studies related to ancillary or non-physician services, such as physical therapy and prescription medications, as access to these services differs in the frequency of use relative to physician services and insurance coverage policies. We did not exclude studies based on their quality, opting instead to include all relevant studies and discuss the strengths and weaknesses as part of the review.

**Figure 1:**
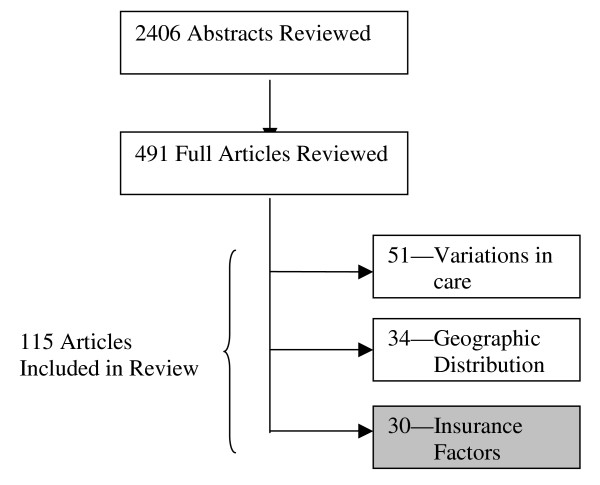
Distribution of articles included in full literature review.

Our initial search strategy identified 2406 abstracts that were potentially relevant to our broader review on access to and quality of specialty care for children (Figure 1). Each abstract was reviewed relative to our exclusion criteria by two reviewers. The senior author (MLM) reviewed disputed abstracts and made the final decision on their inclusion; 491 relevant abstracts were identified for full abstraction. We also reviewed these articles' references for overlooked citations. Two separate reviewers then abstracted each complete article. The primary author (ACS) reviewed all articles to validate their inclusion.

### Definitions

We approached the review with a broad definition of access, aiming to capture all studies that examined how children seek out and use specialty care, which is defined as care delivered by non-primary care physicians. We looked for measures such as utilization, absence of unmet needs, referrals, availability of specialists and appointments, and length of time to referral. Terminology is inconsistent throughout the literature; however, we use consistent terms throughout this review even when they differ from the term used by the original author. Insurance refers to a source of payment for medical services. Uninsured reflects a lack of payment source other than self-pay. Public insurance includes Medicaid and the State Children's Health Insurance Program (SCHIP). Private insurance refers to any coverage that is not publicly funded, including employer-based coverage and individually-purchased coverage. We define a gatekeeping arrangement as one in which patients must visit or receive a referral from a primary care physician before visiting a specialist (i.e., the patient is not able to self-refer without financial consequences). We refer to fee-for-service arrangements as those that reimburse physicians on a per-service basis, rather than a capitated arrangement, and that allow patients to self-refer. We refer to HMOs as those plans that require patients to receive virtually all care within a specified group of physicians, with payment usually made in the form of capitation. The term "managed care" is often used in the literature, referring to insurance of varying types, but typically it requires or provides incentives for using a defined network of providers. If the form of managed care is not evident we refer to this simply as managed care.

### Literature Synthesis

From each included article, we abstracted information on the type of specialty care, research design and methods, sample characteristics, type of comparison (e.g., uninsured vs. insured, Medicaid vs. private, etc.), and findings relevant to the effects of insurance on access to specialty care. Three primary comparisons of the effects of insurance were explored: (1) differences between children with and without insurance, (2) differences between children with public vs. other forms of insurance (generally private), and (3) differences between children in managed care vs. other forms of insurance. Whenever possible, we described characteristics of the insurance coverage. Some articles examined more than one of these comparisons. Substantial variation in methods and measures precluded formal meta-analyses. Therefore, results are presented in narrative form.

## Results

Thirty articles met all inclusion criteria (Tables 1). Most (67%) of the included studies examined all "specialty care," rather than focusing on a single specialty. The remaining studies examined specialists for asthma, juvenile rheumatoid arthritis, cardiac care, urology, and otolaryngology.

**Table 1:** Characteristics of included articles.

*Specialty*	Number	Percent
All/Not specific	20	67
Asthma specialist	6	20
Juvenile rheumatoid arthritis specialist	1	3
Cardiology	1	3
Urology	1	3
Otolaryngology	1	3
*Sample Location*		
Local/Other	12	40
State	12	40
National	6	20
*Access Measure*		
Utilization	17	57
Referral rate	3	10
Referral type	1	3
Satisfaction with availability of specialists	1	3
Unmet need	5	17
Time to referral	1	3
Appointment availability	2	7
*Study Population*		0
Patients	26	87
Physicians	3	10
Both	1	3

### Uninsured

The reviewed studies consistently show a negative association between uninsurance and access to specialty care for children (Table 2). In the five studies that address use of specialty care by uninsured children, these children receive less specialty care than both their privately and publicly insured counterparts [12-16].

**Table 2:** Summary of articles addressing the effects of uninsurance on access to specialty care.

**Author**	**Year**	**Sample Size**	**Data Source**	**Study Design**	**Access Measure**	**Statistic**	**Comparison**	**Findings**	**Endogeneity/Selection**
Kane et al. [12]	2005	700	National Survey of CSHCN, single state	Cross-sectional	Unmet needs	Logistic regression; likelihood of unmet need	Ever uninsured vs. insured all year	OR = 8.6, p < 0.001	No consideration of selection into insurance
Mayer et al. [13]	2003	38,866	National Survey of CSHCN	Cross-sectional	Unmet need	Logistic regression; likelihood of unmet need for specialty care	Private insurance vs. uninsured	OR = 4.29, p < 0.01	No consideration of selection into coverage type
Park et al. [14]	2002	1,985	National Health Interview Survey	Cross-sectional	Utilization	Proportion having seen a specialist (exact values not reported)	Uninsured vs. any insurance type	Less likely vs. any insurance	No consideration of selection into coverage type
Perlstein et al. [15]	1997	544	Regional cardiac registry	Retrospective cohort	Time to referral	t-test; mean age at referral	Uninsured vs. "commercial"	251 days vs. 80 days, p < 0.05	No consideration of selection into coverage type
Szilagyi, et al. [16]	2000	2,126	Single SCHIP	Quasi-experimental	Utilization	t-test, difference in number of specialist visits (pre- and post-enrollment)	Uninsured vs. following SCHIP enrollment	Fivefold increase in utilization after SCHIP enrollment	No consideration of selection in program

The five studies identified on uninsured children found that they use less specialty care and experience greater delays in referrals than do insured children [12-16]. An evaluation of Child Health Plus in New York State showed a five-fold increase in specialist visits before and after enrollment for previously uninsured children [16]. A study of referral delays found that uninsured children had the greatest length of time before referral to a cardiac specialist for congenital heart disease [15]. One national study found that uninsured children with recurrent ear infections were less likely to have a seen a specialist than children with public or private insurance [14]. Another national survey showed that any period of uninsurance is associated with significantly greater unmet needs for care [13], a finding that held when examining results for children in Mississippi [12].

While few studies have examined the effect of being uninsured on children's access to specialty care, many studies have demonstrated that lack of insurance negatively affects access to physician services for children [17-19]. Our findings here are consistent with this previous research. It is unclear if lack of insurance reduces parents' efforts to obtain specialty care for their children or acts as a barrier in instances where people have tried to obtain care.

The lack of insurance may create a two-fold barrier to specialty care, further complicating access for uninsured children. Uninsured children have more difficulty accessing primary care [20], which may be necessary for parents to even realize a need for specialty care. Even if they do receive primary care, the greater expense of specialty care makes it more difficult to obtain than primary care.

On the other hand, uninsured children may use less specialty care because they need less specialty care. None of the studies included here consider selection into insurance plans. Parents may choose to not insure their child if they are confident the child has few needs for health care, particularly the more expensive specialty care.

### Public insurance

Eighteen studies examined differences in access to care for children with Medicaid or SCHIP (Table 3) [13-16,21-33]. Many of these studies suggest that children with public insurance have better or similar access to care, compared to children with private insurance. In a study of pediatricians' referral patterns, children with Medicaid received more specialty referrals than children with private insurance [24]. Similarly, a study of asthmatic children found that children in a state health insurance plan for low-income children and children with Medicaid were more likely to be referred to a specialist than children with private insurance [27]. Children with special health care needs who had public insurance were no more likely than children with private insurance to have unmet needs for specialty care [13].

**Table 3:** Summary of articles addressing the effects of public insurance on access to specialty care.

**Author**	**Year**	**Sample Size**	**Data Source**	**Study Design**	**Access Measure**	**Statistic**	**Comparison**	**Findings**	**Endogeneity/Selection**
Cabana et al. [21]	2002	3,163	Single MCO	Cross-sectional	Utilization	Logistic regression; likelihood of specialty care	Medicaid vs. non-Medicaid insured	Private with copay: OR = 2.52, p < 0.05 Private w/o copay: OR = 3.40, p = NS	Single MCO with Medicaid and private; patients do not choose
Damiano et al. [22]	2003	463	State SCHIP	Prospective cohort	Unmet need	McNemar; unmet need pre- vs. post-enrollment	SCHIP vs. prior coverage	40% vs. 13%; p < 0.05	No consideration of selection into program
Davidoff et al. [23]	2005	3413	National Health Interview Survey	Quasi-experimental	Utilization	Change in proportion with any visit	SCHIP ineligible vs. SCHIP eligible	+3.8, p = NS	Groups compared on eligibility, not enrollment
Forrest et al. [24]	1999	27,104	National practice-based research network	Prospective	Referral rates	t-test, percent referred; logistic regression, likelihood of referral to specialty	Medicaid vs. Private	4.46% vs. 2.61%, p < 0.001	No consideration of selection into coverage type
Holl et al [25]	2000	1,730	Single SCHIP	Quasi-experimental	Utilization	Change in proportion with any specialist visit	Prior to SCHIP enrollment vs. after enrollment	Age < 1 year: 15.5% vs. 16.1%, p = NS; Age 1–5 years: 19.7% vs. 19.4%, p = NS	No consideration of selection into program
Hwang et al. [26]	2005	54	Clinics in a single state	Cross-sectional	Appointment availability	t-test, proportion offering appointment	Private insurance vs. Medicaid	96% vs. 41%, p < 0.0001	Physician offices; no patient selection
Kempe et al. [27]	2000	596	Pediatric practices in a single state	Retrospective cohort	Referral rates	χ^2^; proportion with referral	Private insurance vs. Medicaid	11% vs. 20%, p = 0.09	No consideration of selection into coverage type
Kempe et al [28]	2005	480	Single SCHIP	Prospective cohort	Utilization	Logistic regression; saw specialist when needed; any specialist visit	Prior to enrollment vs. after enrollment	OR = 1.96, p < 0.05; OR = 1.22, p = NS	No consideration of selection into program
Mayer et al. [13]	2004	38,866	National Survey of CSHCN	Cross-sectional	Unmet need	Logistic regression; likelihood of unmet need for specialty care	Private insurance vs. Medicaid and SCHIP	Medicaid: OR = 1.26, p = NS; SCHIP: OR = 0.82, p = NS	No consideration of selection into coverage type
Ortega et al. [29]	2001	1,002	Multiple hospitals; single geographic region	Retrospective cohort	Utilization	χ^2^;percent seeing an asthma specialist	Private insurance vs. Medicaid	30% vs. 6%, p < 0.001	No consideration of selection into coverage type
Park et al. [14]	2002	1,985	National Health Interview Survey	Cross-sectional	Utilization	Proportion having seen a specialist	Private insurance vs. public insurance	Less likely vs. private insurance	No consideration of selection into coverage type
Perlstein et al. [15]	1997	544	Regional cardiac registry	Retrospective cohort	Time to referral	t-test; mean age at referral	Medicaid vs. "commercial"	168 days vs. 80 days, p < 0.05	No consideration of selection into coverage type
Price et al. [34]	1999	94	Single hospital	Cross-sectional	Utilization	t-test; number of specialist visits	Medicaid vs. fee-for-service	All: 3 vs. 6, p = NS; asthma-related: 2 vs.4, p < 0.05	No consideration of selection into coverage type
Szilagyi, et al. [31]	2000	187	Single SCHIP, children with asthma	Quasi-experimental	Utilization	χ^2 ^and t-test; percent seeing specialist, number of visits	Prior to SCHIP enrollment vs. after enrollment	Any specialist: 30% vs. 40%, p = 0.02; Visits: 0.36 vs. 0.48, p = 0.02	No consideration of selection into program
Szilagyi, et al. [16]	2000	2,126	Single SCHIP	Quasi-experimental	Utilization	t-test, difference in number of specialist visits	Prior to SCHIP enrollment vs. after enrollment	0.174 more visits after enrollment, p < 0.001	No consideration of selection into program
Szilagyi et al. [30]	2004	2,644	Single SCHIP	Prospective cohort	Utilization and unmet need	Logistic regression, change in unmet needs pre- and post-enrollment	Prior to SCHIP enrollment vs. after enrollment	15.5 percentage point decrease after enrollment, p < 0.01	No consideration of selection into program
Wang et al. [32]	2004	100	Clinics in single state	Cross-sectional	Appointment availability	Percentage comparisons, no statistical test, percent offering an appointment	Private PPO vs. Medicaid	97% vs. 27%	Physician offices; no patient selection
Zwanziger, et al. [33]	2000	1,910	Single SCHIP	Quasi-experimental	Utilization	OLS, change in expenditures pre- and post-enrollment	Prior to SCHIP enrollment vs. after enrollment	$71.85 increase after enrollment	No consideration of selection into program

Several additional studies also show a positive effect, but do not isolate the effects to compare public coverage to private coverage, instead showing the effects of enrollment into public insurance programs, pooling children who were previously uninsured and those who previous had private insurance coverage. Enrollment in the Iowa SCHIP program significantly reduced unmet need for specialty care compared to the period before enrollment [22]. Children who were eligible for SCHIP at its inception had a small but not statistically significant increase in the likelihood of having any specialist visit [23]. Two studies found that children overall and asthmatic children specifically used more specialty care in their first year with New York State's Child Health Plus coverage, a state-run health program that now includes SCHIP, than in the previous year with another type of coverage, including no insurance [16,31]. Another study of Child Health Plus found that enrollment had a small positive effect on specialty care utilization and accounted for a $.21 average increase in expenditures per child, compared to the child's previous insurance status [33]. Two additional studies of Child Health Plus found no significant difference in utilization of specialty care after enrollment in SCHIP, compared to the year prior to enrollment for all children and children up to age five [25,28]. Finally, enrollment in Child Health Plus was related to significantly fewer unmet needs for specialty care [25,30]. Because these studies group previously uninsured children with those who were insured prior to enrollment we cannot isolate the effects of public insurance by different types of previous coverage.

Although studies suggest that children with public insurance have improved access, particularly if they would otherwise be uninsured, additional studies indicate that they fare worse, compared to privately insured children. Children enrolled in Medicaid managed care organizations were less likely to receive specialty care than children enrolled through non-Medicaid coverage [21]. Similarly, children with "public insurance" were less likely to have seen a specialist than children with private insurance [14]. A study of asthmatic children found that those covered by Medicaid were less likely than children with private insurance to receive specialty asthma care, and when they did, they were less likely to receive it from a board-certified physician [29]. A study of children with severe asthma found that Medicaid-insured children had fewer visits to specialists for sick care or asthma care than children with other forms of insurance [34]. A study that examined referrals from pediatricians found that Medicaid-insured children with congenital heart disease were referred to a pediatric cardiologist at older ages than children with managed care or other private insurance [15]. Two studies that assessed physicians' willingness to accept Medicaid patients showed that children with Medicaid were less likely to be able to get an appointment with either an otolaryngologist [32] or a urologist [26].

The literature investigating the effects of Medicaid or SCHIP coverage on access to specialty care for children is less conclusive than that investigating uninsurance. It is important to note that six of the eleven articles that found either no effect or improvement in specialty care access were samples from the New York State's Child Health Plus program [16,25,28,30,31,33]. Of the seventeen studies that we reviewed, only three studies of public insurance used nationally representative samples [13,14,23]. Therefore, the findings of the studies are not generalizable to Medicaid and SCHIP plans in every state.

Although Medicaid and SCHIP certainly appear to improve access for children who would otherwise be uninsured, Medicaid enrollees appear less likely than children with private coverage to receive a referral to specialty care, to receive specialty care, or to receive that care from a board-certified physician [14,15,21,29,34], and more likely to have difficulties finding a physician willing to accept Medicaid [26,32]. One exception to this is the finding that Medicaid children appear to have more specialty referrals, although these studies do not address whether Medicaid children receive the specialty care to which they are referred [24,27].

The fact that children enrolled in Medicaid and SCHIP have better access then their uninsured counterparts has great importance for current attempts by states to control costs through enrollment freezes, coverage limits, or eligibility reductions [2,3]. Many children may find themselves uninsured due to these policy changes. Lack of coverage, coupled with low incomes, will likely have a significant negative impact on their ability to receive the specialty care they need [13].

One aspect of public insurance not addressed by these studies is the extent to which the scope of covered services affects access to specialty care. Medicaid programs tend to have broad definitions of covered services, while separate SCHIP plans and private insurance plans may have more restrictive guidelines for medical necessity and covered services [35,36]. Therefore, the reason for the greater difficulty accessing care among those with public insurance is not clear. It is possible that it is more difficult for those with Medicaid to find physicians willing to accept Medicaid, or that this population simply faces more barriers in obtaining specialty care, such as transportation, obtaining primary care and its referrals, or recognizing needs for specialty care.

Another important consideration for public insurance programs, often not addressed by current studies, is the endogeneity of public insurance and specialty care. Among the 18 studies that examine public insurance, only four are designed in a way that limits the effects of selection into the Medicaid or SCHIP programs. Specialists often have systems in place to determine if children qualify for public insurance programs and help them obtain coverage. If this is true, children with public insurance may seem to fare relatively well only because they are already under care for a specialist. In addition, publicly insured children may enroll in the programs primarily because their parents know their children need specialty care. The studies consistently show that children use more specialty care after enrollment, which could indicate that the parents seek out coverage specifically due to health care needs. If considering the entire population of children, who do not have prior specific needs for specialty care, the increase in specialty care use might be much smaller.

### Managed care

The findings of the 14 articles that investigated differences in specialty care access between children in managed care arrangements and those in other insurance arrangements were highly inconsistent (Table 4). One set of studies found that managed care arrangements have no effect or a positive effect on access to specialty care. One study found no significant differences in the number of specialty visits for Medicaid-insured infants receiving coverage through a managed care arrangement compared to fee-for-service arrangements [37]. Another reported no differences in use of specialty care for children in Medicaid primary care case management arrangements, compared to traditional fee-for service arrangements [38]. A third found that removal of gatekeeping requirements in a managed care organization resulted in no significant increases in overall utilization of any specialists, although the increase in first-time visits was significant [39]. Children with disabilities are less likely to report an unmet need for a specialist visit if they are in managed care, compared to children in fee-for-service plans [40]. Similarly, another study found that children in a Medicaid HMO were more likely to receive a specialist visit than those in a gatekeeping arrangement, while children in fee-for-service are less likely to receive a specialist visit than the gatekeeping arrangement [41]. A small study of asthmatic children found that those in capitated managed care had more visits to specialists for sick care and asthma care [34]. Pediatricians reported more parental pressure to provide a referral for children in gatekeeping arrangements relative to FFS; however, pediatricians still provided more referrals for children in gatekeeping arrangements after excluding those made at a parent's request [24]. Finally, mechanisms employed by managed care appeared to increase the use of specialty care – there were more specialist visits in an MCO when there were fewer physicians paid on a FFS basis and when a bonus was offered to physicians who met quality of care standards [42], and children in mandatory Medicaid HMO arrangements were more likely to have specialty care visit than those in FFS arrangements [38].

**Table 4:** Summary of articles addressing the effects of managed care on access to specialty care.

**Author**	**Year**	**Sample Size**	**Data Source**	**Study Design**	**Access Measure**	**Statistic**	**Comparison**	**Findings**	**Endogeneity/Selection**
Alessandrini et al. [37]	2001	553	Single hospital	Prospective cohort	Utilization	χ^2^; % with a specialty visit; number of visits	Managed care vs. fee-for-service	10% vs. 12%, p = 0.68; 0.2 vs. 0.2, p = 0.65	MC mandated' no patient selection
Cartland and Yudkowsky [43]	1992	1,264	American Academy of Pediatrics Fellows	Cross-sectional	Referral rates	χ^2^; frequency of referral of MCO patients	Managed care vs. fee-for-service	More frequent: 2.5%; less frequent, 8.7%; p < 0.05	Study is of physician behavior; no patient selection
Cuesta et al. [44]	2000	49	Single hospital	Retrospective cohort	Referral type	χ^2^	Initial referral is to rheumatologist vs. orthopedic surgeon	Managed care: 83% vs. 17%; "Traditional commercial": 58% vs. 42%; p = NS	Examines insurance type at initial referral, prior to diagnosis
Ferris et al. [39]	2002	59,952	Single MCO	Quasi-experimental	Utilization	t-test; number of specialist visits and proportion new specialist visits	With gatekeeping vs. without gatekeeping	Visits: 0.28 vs. 0.28, p = NS; % new visits: 30.6% vs. 34.8%; p < 0.05	Single MCO initiated removal of gatekeeping; no patient choice
Ferris et al. [45]	2001	1,839	Single insurance plan	Prospective cohort	Utilization	t-test; change in visits	Gatekeeping vs. indemnity	57% decrease vs. 31% increase; p = 0.005	Patient voluntarily selected into coverage type
Forrest et al [24]	1999	27,104	National practice-based research network	Prospective	Referral rates	t-test, percent referred; logistic regression, likelihood of referral to specialty	Gatekeeping vs. no gatekeeping	Medicaid, OR = 1.86, p < 0.001; Private, OR = 1.76, p < 0.01	No consideration of selection into type of plan
Garrett et al [38]	2003	34,280	National Health Interview Survey	Retrospective	Utilization	Probit; mandatory PCCM vs. FFS, mandatory HMO vs. FFS; likelihood of any specialist visit	Fee-for-service vs. primary care case management or HMO	PCCM = 0.003, p = NS; HMO = 0.378, p < 0.05	Mandatory enrollment into program type
Lake [46]	1999	12,383	Community Tracking Survey	Cross-sectional	Satisfaction	Logistic regression; difference in percent satisfied with choice of specialists	HMO vs. non-HMO	-8.3%, p < 0.05	No consideration of selection into coverage type
Mitchell, Khatutsky, and Swigonski [40]	2001	966	Single SCHIP	Cross-sectional	Unmet need	χ^2^; percent with unmet need for specialist	Managed care vs. fee-for-service	6.0% vs. 10.6%, p = NS	Patients seek managed care exemptions
Perlstein et al. [15]	1997	544	Regional cardiac registry	Retrospective cohort	Time to referral	t-test; mean age at referral	Managed care vs. "commercial"	140 days vs. 80 days, p < 0.05	No consideration of selection into coverage type
Price et al. [34]	1999	94	Single hospital	Cross-sectional	Utilization	t-test; number of specialist visits	Capitated plan vs. fee-for-service	All: 7.5 vs. 6, p = NS; asthma-related: 5 vs. 4, p,0.05	No consideration of selection into coverage type
Roberto et al. [53]	2005	935	Single Medicaid program	Quasi-experimental	Utilization	Probit; change in access to specialist	Fee-for-service vs. partially capitated managed care	b = 0.221, p < 0.05	Voluntary selection into plan type
Shenkman at al. [42]	2004	2,333	Single SCHIP	Cross-sectional	Utilization	Logistic regression; likelihood of a specialist visit	Plans with certain managed care characteristics vs. those without	Percent paid on FFS basis: 0.950, p = 0.003; Bonus for quality profile: 1.714, p = 0.0003	Mandatory enrollment into specific plan
Shields, et al. [41]	2002	6,231	Single Medicaid program	Cross-sectional	Utilization	Logistic regression; likelihood of specialist visit	HMO vs. primary care case management plan	OR = 1.80, p < 0.05	Voluntary selection into coverage type

Five studies reported that children in managed care arrangements had worse access to specialty care [15,43-46]. One found that 8.7% of pediatricians report referring children in managed care to specialists less than children with fee-for-service insurance [43]. A study of HMO satisfaction reported that families with children in HMOs were less likely than those with other types of coverage to be "very satisfied" with the choice of specialists available to them, which may suggest access barriers [46]. Another study reports that switching to a gatekeeping arrangement resulted in significantly fewer specialist visits for all children. Children with chronic conditions who enrolled in a gatekeeping arrangement had a 57% decrease in specialist use, while children who remained in a FFS arrangement had a 31% increase in specialist use [45]. A small study of children with juvenile rheumatoid arthritis found that those in managed care were more likely to first be referred to an orthopedist rather than a rheumatologist than those in fee-for-service [44]. Another study of physician referral patterns found that children with managed care were older than children with other private insurance at the time of initial referral to cardiac specialist for congenital heart disease [15].

The effects of managed care on access are much less clear than the effects of public insurance. One well-studied aspect of managed care is the use of gatekeeping arrangements, where the child must receive a specialty referral from his or her primary care physician. Gatekeeping arrangement have been found to have positive [24,41], negative [45], and neutral effects [39] on specialty care access. When comparing managed care generally to fee-for-service arrangements, the evidence is also mixed. Children in managed care appear to have later referrals to specialty care, and physicians claim that managed care creates barriers to referrals, including administrative barriers and lack of appropriate care in the plan [41,43,45,46].

There are several potential reasons for the varied results seen for the effects of managed care arrangements on access to specialty care. One previous study showed that families with special needs children are significantly less likely to enroll in gatekeeping plans [47], a finding also seen in one study included here [45]. Thus, the population that might be most susceptible to constraints imposed by gatekeeping selectively avoids these plans. Six of the 14 included studies consider selection bias into managed care plans. Although inconsistency remains, there is a trend towards greater similarities between children enrolled in managed care and those in fee-for-service plans when the plan type or characteristics are mandated, either by changes from the managed care organization or public insurance policies.

Under gatekeeping arrangements, primary care physicians may refer children more because they are required to provide the referral for coverage of the specialty care as opposed to merely recommending self-referral or they feel more pressure from parents to make a referral under these arrangements [24]. Because these studies do not address the issue of the appropriateness of the referral, physicians may also feel time and financial pressures to see as many patients as possible, and use referrals to specialists as a way to divert the care of more complicated and time-consuming patients.

The lack of appropriate care in managed care organizations is of particular concern. Because children in managed care are often limited in which physicians they are allowed to see, they may find themselves unable to obtain the specific type of specialty care needed. Patients in managed care report less satisfaction with the choice of providers available to them [46] and children in managed care with juvenile rheumatoid arthritis appear more likely to be first referred to an orthopedist than a rheumatologist [44]. No other current studies examine the extent to which insurance affects the type of specialty care provider seen or if the cause is limited supply of specialty physicians due to managed care. The literature is peppered with unreferenced comments that certain types of managed care arrangements, such as staff model HMOs and IPAs lack pediatric subspecialty care providers in their networks [48,49]. These comments may reflect evidence that a very small percentage of pediatric subspecialists practice in HMOs [9]. Yet, no other studies have demonstrated differences in the types of subspecialists seen (i.e., adult vs. pediatric subspecialists) by insurance type or explored the effects of the changes in health care financing and organization on the availability of pediatric subspecialty care.

The current attempts by employers to rein in ever-rising health care costs makes it important to understand how their efforts will affect children, particularly those who regularly need specialty care. Although the data are certainly not clear, the use of managed care plans may affect which specialists children can see or require a higher copayment to see their current physician. The move to plans with greater cost-sharing for services, in the form of copayments or premium contributions, will have detrimental effects on children who need care the most, particularly in families with the lowest incomes.

## Discussion

This review provides evidence that the availability and structure of insurance affects children's access to specialty care. However, it also demonstrates that current research is lacking in its ability to show which arrangements best improve the health of children needing specialty care. None of the studies included here explore the relationship between insurance coverage, access to specialty care, and health outcomes.

### Quality of literature

The current literature on children's access to specialty care lacks in many areas. Most of the research uses narrow samples that do not permit generalization to a larger population, do not consider differences in types of specialists or quality of care, do not consider the effects on health outcomes, use limited measures of access to care, and do little to help us understand why there are differences. The existing literature merely demonstrates that differences in access to subspecialty care exist by insurance status.

The primary limitation of current research is the inability to make generalizations across the pediatric population. The studies vary greatly in populations sampled, methods, and means of measuring access to specialty care. For example, studies of managed care effects may be based in a small area, include Medicaid-only populations, or use different definitions of managed care.

The characteristics of current research also leave us unable to answer several important questions. First, the majority of studies that examine access to specialty care for children do not consider differences by insurance in the type of providers used by children, such as a pediatric versus an adult specialist. A study of Medicaid children showed that only a minority of children with serious medical conditions received their care from pediatric subspecialists and that many relied on adult specialists and general pediatricians for their care [50]. The extent to which non-Medicaid children with chronic conditions rely on adult subspecialists and general pediatricians and the relative quality of these providers are important but poorly understood issues.

Another limitation is the lack of information about the child's health outcomes. Although children in gatekeeping arrangements may be more likely to receive a specialty referral, this does not necessarily indicate that they are receiving the most appropriate course of treatment. Similarly, uninsured children may have a higher threshold of illness before they are willing to seek specialty care, but the extent to which such delays actually result in poorer outcomes is not known. Newacheck and colleagues found that while use of managed care by children did lower use of physician services, there was no significant difference in health status [51], although it is not known whether such findings would hold true for children in need of specialty care.

If use of specialty care will not improve a child's health, lower likelihood of referral for certain children is not necessarily a problem. Unfortunately, most research relies on two main outcome measures – physician referral rates and patient utilization. Other measures could improve the ability to make recommendations about what types of insurance provide optimal coverage. One possibility already mentioned is the use of health outcomes. Another possibility is measuring unmet need, the extent to which a child cannot receive care that is needed. Additional information about the symptoms and illness that led to the specialty referral might allow professional judgments of need and appropriateness of referrals.

The research to date also does not allow us to understand the reasons why children with different insurance types have differing access to specialty care. Because so many pediatric specialists are located in academic medical centers, it is likely that they are less sensitive to the price differences between Medicaid, SCHIP, and private insurance. One explanation for this is that insurance coverage is a greater barrier to seeking coverage than actually obtaining it. Insurance type has been shown to affect whether parents of children with special health care needs perceive any need for care at all [52]. Perhaps differences in use of specialty care are caused, in part, by differences in the extent to which parents seek out specialty care or primary care physicians refer children.

We have not considered all health care needs here, only specialty care. The users of specialty care are often, but not always, children with special health care needs. These children frequently need a broad array of services, including physical and occupational therapy, and prescription medications. Because such services are often needed more frequently than physician visits, we would expect the effects of insurance to have a more dramatic effect on these services. Children with mental health conditions may also be particularly vulnerable to the effects of insurance status given the historical lack of parity between coverage for physical and mental health conditions. These are also areas where children with public coverage, particularly Medicaid, fare better as private insurance plans often have limitations of the number of visits covered for mental health visits and other non-physician services.

## Conclusion

Although there is a growing body of literature on the effects of insurance on children's access to specialty care, findings have been so varied that it is not possible to draw firm conclusions about the effects of differing types of insurance. It is clear, however, that children with some form of coverage receive more referrals and make greater use of specialty care than children with no insurance.

This review demonstrates limitations in the current literature that should be pursued in the future. First, additional research is needed on how well Medicaid and SCHIP patients can access specialty care, relative to privately insured children. Second, while overall utilization and referral rates are useful starting points for studying access to specialty care, it is also important to understand how insurance affects the types of specialists seen. Finally, future research should address how these differences affect health outcomes.

Based on the current literature, we cannot determine the structure of insurance that leads to the best use of appropriate, quality care by children. Only with additional research about the specific characteristics of health plans and the effects on health outcomes will it be possible to determine a structure of insurance coverage that provides optimal access to specialty care for children.

## Competing interests

The author(s) declare that they have no competing interests.

## Authors' contributions

ACS carried out the literature review, synthesized the literature, and drafted and revised the manuscript. MLM conceived of the idea, carried out the literature review, synthesized the literature, and assisted in revising the manuscript. Both authors read and approved the final manuscript.

## Pre-publication history

The pre-publication history for this paper can be accessed here:



## Supplementary Material

Additional file 1 Search criteria. Detailed description of the search criteria used in the literature review. Click here for file
