# Supplementary material for: Effects of insurance status on children's access to specialty care: a systematic review of the literature
Source: BMC Health Serv Res. 2007 Nov 28;7:194. doi: 10.1186/1472-6963-7-194 (PMC2222624; doi:10.1186/1472-6963-7-194)
Supplement: Additional file 1 — Search criteria. Detailed description of the search criteria used in the literature review. [file 1472-6963-7-194-S1.doc]

Search Criteria.

|  | ***and*** | ***And*** | ***And*** |
| --- | --- | --- | --- |
| **MeSH** | **MeSH** | **MeSH or Keywords** | **Limits** |
| Specialty care  Medical specialties  [Allergy and Immunology](http://www.ncbi.nlm.nih.gov:80/entrez/query.fcgi?cmd=Retrieve&db=mesh&list_uids=68000486&dopt=Full)  Anesthesiology  [Dermatology](http://www.ncbi.nlm.nih.gov:80/entrez/query.fcgi?cmd=Retrieve&db=mesh&list_uids=68003880&dopt=Full)  [Emergency Medicine](http://www.ncbi.nlm.nih.gov:80/entrez/query.fcgi?cmd=Retrieve&db=mesh&list_uids=68004635&dopt=Full)  [Internal Medicine](http://www.ncbi.nlm.nih.gov:80/entrez/query.fcgi?cmd=Retrieve&db=mesh&list_uids=68007388&dopt=Full)  [Cardiology](http://www.ncbi.nlm.nih.gov:80/entrez/query.fcgi?cmd=Retrieve&db=mesh&list_uids=68002309&dopt=Full)  [Endocrinology](http://www.ncbi.nlm.nih.gov:80/entrez/query.fcgi?cmd=Retrieve&db=mesh&list_uids=68004704&dopt=Full)  [Gastroenterology](http://www.ncbi.nlm.nih.gov:80/entrez/query.fcgi?cmd=Retrieve&db=mesh&list_uids=68005762&dopt=Full)  [Hematology](http://www.ncbi.nlm.nih.gov:80/entrez/query.fcgi?cmd=Retrieve&db=mesh&list_uids=68006405&dopt=Full)  [Medical Oncology](http://www.ncbi.nlm.nih.gov:80/entrez/query.fcgi?cmd=Retrieve&db=mesh&list_uids=68008495&dopt=Full)  [Nephrology](http://www.ncbi.nlm.nih.gov:80/entrez/query.fcgi?cmd=Retrieve&db=mesh&list_uids=68009398&dopt=Full)  [Pulmonary Disease (Specialty)](http://www.ncbi.nlm.nih.gov:80/entrez/query.fcgi?cmd=Retrieve&db=mesh&list_uids=68015272&dopt=Full)  [Rheumatology](http://www.ncbi.nlm.nih.gov:80/entrez/query.fcgi?cmd=Retrieve&db=mesh&list_uids=68012219&dopt=Full)  Neonatology  [Neurology](http://www.ncbi.nlm.nih.gov:80/entrez/query.fcgi?cmd=Retrieve&db=mesh&list_uids=68009462&dopt=Full)  Pediatrics  Neonatology  [Radiology](http://www.ncbi.nlm.nih.gov:80/entrez/query.fcgi?cmd=Retrieve&db=mesh&list_uids=68011871&dopt=Full)  Surgical specialties  Colorectal surgery  [Neurosurgery](http://www.ncbi.nlm.nih.gov:80/entrez/query.fcgi?cmd=Retrieve&db=mesh&list_uids=68009493&dopt=Full)  [Ophthalmology](http://www.ncbi.nlm.nih.gov:80/entrez/query.fcgi?cmd=Retrieve&db=mesh&list_uids=68009885&dopt=Full)  [Otolaryngology](http://www.ncbi.nlm.nih.gov:80/entrez/query.fcgi?cmd=Retrieve&db=mesh&list_uids=68010036&dopt=Full)  [Surgery](http://www.ncbi.nlm.nih.gov:80/entrez/query.fcgi?cmd=Retrieve&db=mesh&list_uids=68013502&dopt=Full)  [Surgery, Plastic](http://www.ncbi.nlm.nih.gov:80/entrez/query.fcgi?cmd=Retrieve&db=mesh&list_uids=68013518&dopt=Full)  [Thoracic Surgery](http://www.ncbi.nlm.nih.gov:80/entrez/query.fcgi?cmd=Retrieve&db=mesh&list_uids=68013903&dopt=Full)  [Urology](http://www.ncbi.nlm.nih.gov:80/entrez/query.fcgi?cmd=Retrieve&db=mesh&list_uids=68014572&dopt=Full) | Pediatrics  Infant  Child  Adolescent | Comparative study  Managed care  Health services accessibility  Manpower  Health Maintenance Organizations  Insurance  Physician practice patterns  Referral and consultation | Human  English  All child: 0-18  Dates:  January 1, 1992–July 31, 2006. |
